# Editorial: Imaging Approaches to Unravel Chromatin Organization and Nuclear Dynamics

**DOI:** 10.3389/fmolb.2022.929370

**Published:** 2022-05-20

**Authors:** Hendrik Sielaff, Srinjan Basu, Ziqing Winston Zhao

**Affiliations:** ^1^ Department of Chemistry, Faculty of Science, National University of Singapore, Singapore, Singapore; ^2^ Centre for BioImaging Sciences (CBIS), Faculty of Science, National University of Singapore, Singapore, Singapore; ^3^ Wellcome Trust - Medical Research Council Cambridge Stem Cell Institute, School of Biological Sciences, University of Cambridge, Cambridge, United Kingdom; ^4^ Department of Physiology, Development and Neuroscience, Faculty of Biology, School of Biological Sciences, University of Cambridge, Cambridge, United Kingdom; ^5^ Mechanobiology Institute (MBI), National University of Singapore, Singapore, Singapore

**Keywords:** chromatin organization, nuclear dynamics, phase separation, enhancer-promoter communication, computational modelling, single-cell imaging, telomere, imaging

Chromatin plays a central role in the maintenance and propagation of genetic information in the eukaryotic cell, impacting the access, expression, duplication, and repair of the genome through interacting with numerous molecular players in the cell nucleus. In addition to their biochemistry, the organization and dynamics of these diverse molecular interactions within the cell nuclear “space-time” are equally critical in regulating the outcomes of chromatin-based transactions. Recent key developments in quantitative imaging- and Hi-C based strategies for probing chromatin organization and dynamics *in cellulo* have played an especially crucial role in reshaping our understanding of the multiscale nuclear architecture in both physical and sequence space. These experimental approaches have in turn been complemented by *in silico* modeling based on concepts from soft matter and polymer physics, hence establishing rigorous theoretical underpinnings for the various spatio-temporal features and patterns observed for these processes.

In this Research Topic, we amass a unique collection of interrelated studies which shed light on key aspects of chromatin organization and nuclear dynamics in both space and time from experimental, computational and conceptual perspectives. In the first article, Vivante et al. focus on lamins, which are known to not only provide structural stability for the nuclear lamina but also play important roles in chromatin organization. By exploring the live-cell localization of lamin A and its dynamic interactions with chromatin across the cell cycle, they revealed a phosphorylation-dependent disassembly of the nuclear lamina and redistribution of lamin A from chromosomal regions to the entire cytoplasm with the onset of mitosis. However, upon entering the telophase and early G1, lamin A transitions from a state where it spreads over the whole volume of the newly formed nuclei of the daughter cells to one that assembles on and binds to the nuclear lamina, with increased concentration on chromatin during the S phase. These previously unknown phenomena offer novel mechanistic insights into the dynamic interplay between lamin and chromatin and its potential functional implications during the cell cycle.

On the methodological front, Xu et al. developed a novel tool for visualizing, tracking and manipulating telomeric repeat-containing RNAs (TERRAs), a type of long non-coding RNAs transcribed from telomeres that are critical for telomere integrity and maintenance, but for which the mechanism of how they regulate telomere functions at the subcellular level remains unclear. By combining CRISPR/Cas13-based RNA-labeling with SunTag technology, their new method provides a general method to detect endogenous TERRA molecules with a regular confocal microscope, while enabling long-term imaging of TERRA dynamics in live cells as a consequence of increased photobleaching resistance provided by the SunTag system. Collectively, these advantages make for a powerful tool for probing TERRAs localization to better understand their roles in telomere maintenance and genomic integrity, while also allowing it to be used to study phase-separation phenomena involving TERRAs, telomeres and the alternative lengthening of telomeres (ALT) pathway.

Indeed, liquid-liquid phase separation (LLPS) has recently emerged as a prominent theme in the field of chromatin organization and nuclear dynamics, especially in light of the fact that the eukaryotic cell nucleus is highly crowded and heterogeneous, which makes membraneless compartmentalization a highly effective strategy for organizing the intranuclear environment and regulating diverse chromatin-based processes, particularly gene expression. In addition to growing experimental evidences on LLPS-mediated chromatin dynamics acquired from a wide range of biological systems, multi-scale computational models have also been developed to provide the theoretical foundations for these newly observed chromatin-based compartments. Here, Laghmach et al. review these emerging computational models, including those based on equilibrium polymer simulations and hybrid non-equilibrium simulations as well as mesoscopic field-theoretic models. By illustrating how these models can make concrete predictions about various biophysical properties of chromatin condensates, this work not only provides sound theoretical paradigms for understanding the roles of phase separation in regulating nuclear organization and dynamics, but also points to the need for an integrated approach that combines quantitative imaging, mesoscale modeling and machine learning-based data analysis in order to achieve a more thorough and accurate understanding of these phenomena.

Finally, chromatin organization is also intricately linked with gene expression and its regulation *in vivo*. To that end, an open question in the field is how enhancers and promoters communicate to control and activate transcription during tissue development. Although enhancer-promoter communication was originally thought to require physical interactions or proximity, recent live-cell imaging studies indicate that this is not always the case. Wurmser and Basu revisit the nature of enhancer-promoter communication, both in the context of LLPS as well as in recent studies that argue against a simple contact-based transcriptional activation. They offer valuable perspectives on some of the key questions the community must still address if we were to provide reliable models of enhancer-promoter communication. Importantly, with the growing use of DNA proximity-based technologies, this work also provides a timely re-conceptualization of whether and how enhancer-promoter proximity relates to transcription.

In summary, by covering a broad range of topics at experimental, computational and conceptual levels, this Research Topic highlights the multiscale and multifaceted complexities of chromatin organization and nuclear dynamics. It is foreseeable that the continuous intermingling between these complementary approaches will lead to better strategies for unraveling such complexities, hence generating more insights into the biochemistry, biophysics and pathology of chromatin-based nuclear processes in years to come.

